# Why Do Patients Miss Dental Appointments in Eastern Province Military Hospitals, Kingdom of Saudi Arabia?

**DOI:** 10.7759/cureus.2355

**Published:** 2018-03-21

**Authors:** Ambreen Shabbir, Mohammad Alzahrani, Areej Abu Khalid

**Affiliations:** 1 Pathology, Prince Sultan Military College of Health Sciences, Dhahran, Kingdom of Saudi Arabia; 2 Vice Dean for Development and Quality, Department of Dental and Oral Health, Prince Sultan Military College of Health Sciences, Dhahran, Kingdom of Saudi Arabia; 3 Chairperson, Department of Dental and Oral Health, Prince Sultan Military College of Health Sciences, Dhahran, Kingdom of Saudi Arabia

**Keywords:** missed dental appointments, eastern province military hospital, kingdom of saudi arabia

## Abstract

No-shows for scheduled appointments are a frequent occurrence, creating unused appointment slots and reducing patient quality of care and access to services while increasing loss to follow-up and medical costs. The aim of our study was to determine the factors that lead to patients missing their dental appointments in Eastern Province Military Hospitals, Kingdom of Saudi Arabia. The study population included military personnel and their families attending the dental clinics of these hospitals. In our study, the percentage of missed appointments was 58.1%, while 54.4% of participants canceled dental appointments in the past. Thirty-six percent preferred morning appointments while 56% preferred an afternoon appointment and were likely to miss a morning appointment if given one. The most common reasons for missing an appointment were forgetting about it (24.3%) and the inability to get time off either from work or school (15.4%); 1.5% of patients stated they had a bad dental experience and feared dental treatment while the unavailability of transport accounted for 0.7% of patients. Of the reasons given for canceling an appointment, the inability to get time off from work/school was the most common (22.1%) while a dislike for treatment was the least common (0.7%). Canceling an appointment was significantly correlated with missing an appointment among the surveyed sample (P=0.00). In our research, 60.3% of participants still relied on their personal diary to remember appointments, which could be a reason for the high rate of missed appointments. Fifty-nine percent of respondents felt that missing an appointment was important to them, while 72% stated that missed appointments could affect the work of the clinic but still believed that automatic appointments should be given to patients who missed them and a change be made accordingly. Since major factors included a lack of a reminder message and appointments scheduled at inconvenient timings, some steps that can help reduce the frequency of missed appointments include sending a reminder message to patients, giving preference to their schedules for appointments, giving patients shorter appointments, reducing intervals between subsequent appointments, and educating patients regarding the treatment plan, to reduce anxiety.

## Introduction

Although general medicine and surgery suffer from non-attendance, it is especially prevalent in dental practice [1], causing detrimental effects on its outcome and revenue [2-3]. Patients may miss their dental appointments for several reasons. Even if the reason is justifiable from the patient’s point of view, it still has a negative impact on the clinic, which eventually trickles down to the entire health system. The impact of missed dental appointments is not as simple as it appears, as the patient not only denies dental care to himself but others as well [4]. At the same time, they interrupt their continuity of care, affect workflow, waste resources, and reduce population-wide access to care [1,5-6].

The economic effect alone of dental patients missing their appointments was estimated at around 65 pounds per appointment according to a study conducted in the United Kingdom in 1997. The same study stated that out of 14-million patients seen in outpatient dental clinics per year, the national rate of nonattendance at appointments was around 12%, which cost the health ministry around 300 million pounds per year [7]. The resources employed by the dental clinic remain idle and underutilized while the dentist waits for the patient to show up and the next patient in line has to wait longer [3]. From the patient’s perspective, if the diagnosis of a particular problem is delayed due to a missed appointment, it delays treatment, thus threatening the patient’s dental health [6].

Most investigations related to causal factors for missing dental appointments have had low response rates (30% to 40%) and are difficult to interpret [7]. However, some common reasons that previous studies have unearthed include unforeseen circumstances, fear of dental treatment, lack of travel facilities, laziness, and forgetfulness [4]. The average non-attendance rate at outpatient clinics in the United Kingdom was reported to be 12%, out of which 30% claimed forgetfulness and 8% no longer felt the need for treatment. One participant revealed that he failed to attend his appointment due to a fear of being seen by a junior doctor, who he believed was inexperienced, while another participant pointed out that he was previously mistreated by the management at the clinic [7]. Similar research conducted at the Kuwait University Dental Center suggested that most no-show dental appointments were significantly influenced by the complexity of the treatment planned, concluding that fear plays an important role in this regard, as most patients were afraid of complex procedures (root canals and extractions), the frequency of which is relatively low compared to regular check-ups [8].

Many patients in Saudi Arabia miss dental appointments each year, which has a significant impact on its health care system as well as the individual clinics [6]. No-shows not only reduce access to care but also interrupt the continuity of care and effective disease management for patients [1]. Studies conducted previously in Saudi Arabia focused more on the consequences of missed dental appointments and concentrated on college students and demographics such as gender. However, to devise a solution to this problem, it must be understood from a broader perspective in Saudi Arabia [6]. This research aimed at finding out the factors responsible for patients missing their dental appointments so preventive steps could be taken in order to reduce its devastating consequences in Eastern Province Military Hospitals, Kingdom of Saudi Arabia.

## Materials and methods

The study was conducted after receiving approval from the Research Ethics Committee, Prince Sultan Military College of Health Sciences, in Dhahran. Our target population was military officers and their families who came for their dental checkups and treatment at Armed Forces Hospital in Jubail, King Fahd Military Medical Complex in Dhahran, and Air Base Hospital in Dhahran. A total of 150 patients visiting the dental clinics were selected at random. The method for collecting data was through a self-administered questionnaire (Appendix A), which was used by researchers at King Saud University in Riyadh [9] after obtaining permission from them and was developed according to the questions mentioned in the article. For those patients who preferred answering in Arabic, the questionnaire was translated into Arabic as well. The questionnaires were handed out to patients at different times of the day and were collected once filled out. Options in the questionnaire had several category choices of "yes/no/do not know." A tickbox layout was used to provide appropriate answers. After collecting the questionnaires from the patients, the answers were coded and entered into an Excel sheet and submitted to a statistician for analysis. The distributions of all qualitative variables (i.e., closed-ended)/values of the sample were examined with frequency tables. Comparison tables were calculated to find an association wherever necessary, and for comparing variables, we used the chi-square test and McNemar’s test.

Inclusion criteria

Literate patients (male/female) who are attending military hospitals in the Eastern Province were included in this study.

Exclusion criteria

Dental staff and patients who could not read or write were excluded from the study.

## Results

A total of 150 questionnaires were distributed, of which 136 were received, giving us a response rate of 90.6%. Thirty-six percent of participants were males, 64% were females, 35.3% were students, and 46.3% were employed, as depicted in Table 1.

**Table 1:** Distribution of the sample by age, gender, and occupation

Demographic variable		
Gender	Frequency	%
Male	49	36
Female	87	64
Occupation	Frequency	%
Student	48	35.3
Employed	63	46.3
Age Group	Frequency	%
12–15 years	5	3.6
16–19 years	22	16
>20 years	110	80

We found that 60.3% respondents used their diaries and 5.9% relied on their memory for remembering their dental appointments, as seen in Table 2.

**Table 2:** Distribution of the sample by methods used for remembering appointments

Method of Remembering Dental Appointment Date	Frequency	%
Diary	82	60.3
Calendar	14	10.3
Memory	8	5.9
Another person	4	2.9
Mobile	2	1.5

When asked for the reasons why they previously canceled their dental appointments, 22.1% patients answered that they were unable to get time off from work/school and 24.3% admitted to forgetting their appointment, as tabulated in Table 3.

**Table 3:** Patients’ reasons for canceling and missing dental appointments

Reason for Cancelation of the appointment	Number	%
Inconvenient timing	8	5.9
Unable to get off work/school	30	22.1
Unable to get transport	12	8.8
Sickness	2	1.5
Fear of treatment	2	1.5
Dislike of treatment	1	0.7
Reason for missing the appointment		
Forgetfulness	33	24.3
Unable to get time off work	21	15.4
School	7	5.1
Unable to get transport	1	0.7
Sickness	2	1.5
Fear of treatment	2	1.5

The association between missed and cancelled appointments is shown in Table 4, with chi-square = 14.10 (p = 0.00). The associations between cancelled appointments and gender, missed appointments and gender, and missed appointments and age group were calculated but did not give a significant p-value (chi-square = 0.22, p = 0.64; chi-square = 1.57, p = 0.21; chi-square = 1.79, p = 0.41, respectively).

**Table 4:** Association between missed and canceled appointments chi-square = 14.10, p = 0.00

		Have you ever missed an appointment at this clinic?		
		Yes	No	Total
Have you ever canceled an appointment at this clinic?	Yes	54	20	74
		73.0%	27.0%	100.0%
	No	25	36	61
		41.0%	59.0%	100.0%
	Total	79	56	135
		58.5%	41.5%	100.0%

We recorded the participants' responses about the consequences of missed appointments in Table 5.

**Table 5:** Response to the consequences of missed appointments among the surveyed sample

Should Another Appointment Automatically Be Sent?	Patients Who Missed an Appointment	Patients Who Never Missed an Appointment	Total
Yes 106	79	57	136
No 30	56	80	136
Do not know 0	0	0	
Total 136	136	136	
When Should an Appointment Be Sent?	Frequency	%	
No answer provided	33	24	
After the first missed appointment	69	50.3	
After every missed appointment	35	25.5	
Total	137	99.5	
If you fail to attend an appointment without prior notification does it matter to you?	Yes	No	Don’t know
	81	29	26
Do you think missing an appointment affects the clinic?	Yes	No	Don’t know
	99	12	24

Seventy-nine patients who had previously missed a dental appointment believed that another appointment should automatically be given to a patient once he/she misses an appointment, while 80 patients who never missed an appointment answered in the negative. When asked if missing an appointment affects the clinic, 99 patients who had previously missed an appointment answered in the affirmative, while only 12 respondents who had never missed dental appointments said it did not affect the clinic.

## Discussion

No-shows for scheduled appointments are a frequent occurrence, creating unused appointment slots, reducing patient quality of care and access to services, while increasing loss to follow-up and medical costs [1]. The study purpose was identifying factors that lead to patients missing their dental appointments in Eastern Province Military Hospitals, Dhahran. In our research, the percentage of missed appointments was found to be 58.1%, which is higher than the 24.8% reported in a study conducted in Riyadh, Saudi Arabia [9] and the 36.8% reported in a study in India [4]. Our study calculated 54.4% of the participants who canceled dental appointments in the past, which is higher than the 40.5% in Dr. Salwa Alsadhan’s study [9]. Out of our study population, 36% of patients preferred morning appointments, while 56% preferred afternoon appointments, which means that if these patients (56%) were given a morning appointment, they would most likely miss it, as their preference was an afternoon appointment.

The most common reasons for missing an appointment were forgetting about it (24.3%) and the inability to get time off either from work or school (15.4%), which is consistent with the ﬁndings of most of the previous studies [9-13]. In our research, we found that only 1.5% of patients feared dental treatment, while the unavailability of transport accounted for 0.7% of missed appointments. Dr. Salwa Alsadhan's study identified 9.1% of patients claiming lack of transport as the reason for missing their dental appointment [9]. For canceling an appointment, inability to get time off from work/school was the most common reason (22.1%), which is similar to a number of studies [9,14] but different from a study done in 1991, which stated illness to be the most common reason [15]. Dislike of treatment for the cancelation of an appointment was the least common (0.7%) in our study while being most prevalent in a study conducted in the United Arab Emirates [16]. The association between canceling and missing an appointment (Table 4) was found to be statistically signiﬁcant (p=0.00), which might indicate that patients who canceled their appointments were more likely to miss an appointment (73%) compared to patients who never canceled their appointments (41%). In our research, 60.3% of participants still relied on their personal diary to remember appointments (Table 2), which could be a reason for the high rate of missed appointments, as 24.3% stated they forgot their appointment (Table 3). Studies suggested a high percentage of individuals making a mental note of their dental appointments [9]. It would be reasonable to assume that missed appointment rates could be reduced if patients were advised to use their mobile phones/computers to record and remember their appointments and if reminder messages/confirmation calls were sent to patients a day prior to their appointment [2,4,9,11]. However, some studies point out that the effect of reminder calls is not confirmatory [12], thus underlining the importance of further, large sample studies.

In our study, females were found to cancel and miss dental appointments more than males although the differences were insignificant (p = 0.64 and p = 0.21, respectively). These numbers differ from a study [17] that suggested males having a higher missed appointment frequency while others suggested females miss more appointments [14].

Fifty-nine percent of respondents felt that missing an appointment was important to them, while 72% stated that it could affect the work of the clinic but still believed that automatic appointments should be given to patients who missed their appointments and a charge be made for such missed appointments (Table 5), which has been suggested by some researchers [18-19].

In dental practice, missing appointments can disrupt the patients’ treatment to a large extent. For example, orthodontic treatment relies on regularly adjusting appliances and monitoring the progress of occlusal changes, whereas the early detection and treatment of carious lesions and gingival and periodontal disease demand regular dental visits, and missing appointments can seriously limit treatment efficiency. Therefore, to control missed and canceled appointments, the dental team must educate the patient on their first visit and cautiously communicate the importance of maintaining the appointment schedule and its effect on treatment outcomes. The patient should also be told how and when they can inform the clinic in case they were unable to make it to their appointment [20].

Future studies should focus on the methods that can reduce or eliminate missed and canceled appointments and encourage patients’ attendance in order to enhance the treatment outcome in addition to improving the economics and quality of dental practice.

## Conclusions

Our study identified a high percentage of patients who miss their dental appointments in the Eastern Province Military Hospitals, Kingdom of Saudi Arabia, causing not only detrimental effects for the patient community but also a wastage of resources at military hospitals. Since the major reasons found were a lack of reminder message and appointments scheduled at inconvenient timings, steps to reduce the frequency of missed appointments should include sending reminder messages to patients, giving preference to their schedules for appointments, giving them shorter appointments, reducing the intervals between subsequent appointments, and educating them regarding the treatment plan to reduce anxiety. Our study sample was small and centered around military hospitals, which may not be representative of the entire population. New studies should be conducted with a larger representative sample size to investigate factors responsible for no-shows so that steps can be taken to prevent them.

## Appendices

Appendix A

C O N F I D E N T I A L

Dear Patients:

Please take a few minutes to answer this questionnaire about the following study titled

‘‘Why Patients Miss Dental Appointments in the Eastern Province Military Hospitals, Kingdom of Saudi Arabia.”

“Participation is optional and opting not to participate in this survey shall not in any way affect your professional status or relationship with the students. The intent of this survey is harmless and the information provided will remain strictly confidential and will never be used for purposes other than the intended purpose of this study.

Thank you for your cooperation.

Principal Investigators:

Dr. Ambreen Shabbir

Lt. Col./Dr. Mohammad Al Zahrani

Prince Sultan Military College of Health Sciences,

Department of Dental and Oral Health,

Dhahran, 31932

Cell phone 966556966683

Phone 966-3-8440000, Ex. 6756

Fax# 966-3-840-5577

Questionnaire:

We would be grateful if you would take a few minutes and complete this questionnaire.

Please tick the appropriate box.

1.  Age: ______________________

2.  Gender:               Male                   Female

3.  Occupation:      Student                 Employee              Other (specify)      ____________

4.  Education:        Illiterate                Elementary            Intermediate

     Secondary             University and above

5. How do you remember your appointment date?

Diary

Mobile

Calendar

Memory

Another person

Other (specify) ­­­­­­­­______________________________________________________

6. Have you ever had to cancel an appointment at this clinic?            Yes               No

If the answer is no to Q6, please skip Q7.

7. Reasons for canceling an appointment (you can choose more than one answer)

Inconvenient timing

Unable to get off work/school

Unable to get transport

Sickness

Fear of treatment

Dislike of treatment

Other reasons (please specify) _______________________________________

8. Have you ever failed to attend an appointment at this clinic without prior notification?              Yes           No

If the answer is no to Q8 then please skip Q9.

9. Reasons for failing to attend an appointment (you can choose more than one answer)

Forgetfulness

Unable to get time from work/school

Unable to get transport

Sickness

Fear of treatment

Dislike toward treatment

Other reasons (specify)_____________________________

10. If a patient fails to attend an appointment, do you think that another appointment should

automatically be sent to them?             Yes             No

11. If yes, when do you recommend should an appointment be sent?

After the first missed appointment

After every missed appointment

12. If you fail to attend an appointment without prior notification:

Does it matter to you?                        Yes           No         Don’t know

Do you think it affects the clinic?      Yes          No           Don’t know

13. If you need to change an appointment, how much notice do you think should be acceptable to the hospital/clinic?

At least 24 hours before appointment time

Less than a week

One week

More than a week

14. What is your preferred time for attending an appointment?

Early morning

Late morning

Early afternoon

Late afternoon

No preference

15. How do you think a patient who misses his/her appointment should be dealt with?

Do not give him/her new appointment before 6 months.

Put him/her on the waiting list.

They should not be given any further appointments at all.

Thank you for your cooperation in completing this questionnaire.
